# 基于pH响应涂层柱的毛细管电泳法在线富集和分离糖蛋白

**DOI:** 10.3724/SP.J.1123.2023.09021

**Published:** 2024-09-08

**Authors:** Jian ZHANG, Caifeng XU, Cailian MA, Maofang HE, Bo ZHANG, Chunye LIU

**Affiliations:** 西安医学院药学院,陕西西安 710021; School of Pharmacy, Xi’an Medical University, Xi’an 710021, China

**Keywords:** 毛细管电泳, 在线富集, 离子吸附, 糖蛋白, 苯硼酸, capillary electrophoresis (CE), on-line enrichment, ion adsorption, glycoprotein, phenylboronic acid

## Abstract

本研究通过静电自组装法制备了一种3-氨基苯硼酸(APBA)修饰的纳米金(AuNPs@APBA)涂层毛细管柱,该毛细管柱兼具抗非特异性吸附性和富集选择性,将其作为分离通道,建立了基于pH响应涂层柱的糖蛋白在线富集与分离方法。首先,采用柠檬酸钠还原法制备金纳米颗粒(AuNPs),再将APBA通过静电自组装修饰到AuNPs表面,制得AuNPs@APBA纳米材料;利用离子吸附作用将AuNPs@APBA吸附到毛细管内壁上,制得AuNPs@APBA涂层毛细管柱。在pH为8的条件下,糖蛋白能够与毛细管内表面的硼酸基团结合形成硼酸酯,从而被吸附;当pH为3时,硼酸酯发生解离并释放出糖蛋白,基于上述原理,该方法能够实现糖蛋白的选择性在线富集与分离。实验结果表明,与使用普通电泳法的裸柱相比,经AuNPs@APBA涂层毛细管柱富集后,卵清蛋白(OVA)的峰面积增大了26.46倍,而牛血清白蛋白(BSA)的峰面积仅增大了8.47倍;并且,将鸡蛋清样品稀释1×10^6^倍后,仍可利用该方法对糖蛋白进行检测。该方法样品用量少,操作简单,分离效率高,可用于实际样品中痕量糖蛋白的在线富集与分离。

## Abstract (en)

A capillary column coated with 3-aminophenylboronic acid (APBA)-functionalized gold nanoparticles (AuNPs@APBA) was prepared via electrostatic self-assembly. The coated column exhibited anti-nonspecific adsorption of glycoproteins, enabling selective online enrichment during capillary electrophoresis (CE). First, gold nanoparticles (AuNPs) were synthesized using the sodium citrate reduction method. Then, APBA was self-assembled electrostatically on the surface of the AuNPs to obtain AuNPs@APBA. This nanomaterial was bonded to the inner wall of a capillary through ion adsorption to produce a AuNPs@APBA-coated capillary column. Glycoproteins were adsorbed via bond formation with boric acid groups under alkaline conditions (pH 8) to generate borate esters. Under acidic conditions (pH 3), the borate esters dissociated to release the glycoproteins, thereby achieving the selective online enrichment and separation of glycoproteins. The AuNPs and AuNPs@APBA were characterized using Fourier transform infrared spectroscopy, and their sizes and Zeta potentials were determined. In addition, the electroosmotic flow (EOF) of the AuNPs@APBA-coated capillary column was measured. The results showed that the surface of the AuNPs was successfully modified with APBA and that AuNPs@APBA was adsorbed on the inner wall of the capillary. The peak area of ovalbumin (OVA) on the AuNPs@APBA-coated column was 26.46 times higher than that on a bare column via conventional electrophoresis. In contrast, the peak area of bovine serum albumin (BSA) only increased by 8.47 times, indicating that the AuNPs@APBA coated column selectively enriched glycoproteins. Evaluation of the reproducibility and stability of this method revealed that the AuNPs@APBA coated capillary column could be used continually for 33-67 h. The relative standard deviations (RSDs) of the peak areas for intra-day (*n*=5) and inter-day (*n*=6) analyses were 2.2% and 3.0%, respectively. The developed method was successfully applied to enrich glycoproteins in a 1×10^6^-fold diluted egg white sample. Glycoproteins were not detected using conventional electrophoresis on the bare column, whereas the AuNPs@APBA-coated capillary column effectively enriched and separated glycoproteins, resulting in a peak area of 10469 mAU·ms. Furthermore, the entire enrichment and separation process was completed within 3 min. This new online enrichment and separation method for glycoproteins has the advantages of low sample consumption, simple operation, and high separation efficiency.

糖蛋白是生物体内重要的功能蛋白,主要包括转录因子、染色质结合蛋白、RNA聚合酶Ⅱ、核孔蛋白、蛋白质翻译调控因子等。糖蛋白不仅具有独特的理化性质,还参与多项生理活动;同时,糖蛋白在病原对宿主的感染、癌症的发生和转移等生物过程中也起着重要作用,这使其成为癌症预后和诊断的重要靶点^[1,2]^。此外,不同细胞阶段表达的糖蛋白水平及糖蛋白类型的改变与许多人类疾病相关^[3]^。因此,针对蛋白质糖基化过程的研究不仅有利于加深对糖蛋白生物功能的认识,而且对探讨疾病发生、发现疾病标记物^[4,5]^和开发新药均具有重要意义。

糖蛋白在生物样品中的含量较低,对糖蛋白的含量进行测定时会同时受到其他高丰度组分的干扰,因此建立痕量糖蛋白的高选择性分离富集方法,对糖蛋白的研究至关重要^[6]^。针对糖蛋白结构中的糖链或其本身的理化性质,现有的富集策略主要包括凝集素亲和色谱法^[7]^、硼亲和色谱法^[8,9]^、肼化学方法^[10]^和亲水作用色谱法(hydrophilic interaction chromatography, HILIC)^[11]^。硼亲和色谱法是糖蛋白分离富集的有效方法之一,其可通过改变pH来实现糖蛋白的选择性吸附与释放,但该方法存在试剂用量大、分析耗时久等问题。毛细管电泳(CE)具有分析速度快、试剂用量小、分离效率高等优点,已广泛应用于蛋白质的分离分析^[12,13]^,但将CE用于糖蛋白的分离分析却报道较少^[14,15]^,并且用于糖蛋白富集毛细管柱的制备步骤繁琐,耗时较长(>30 h)^[14]^。针对以上情况,将硼亲和色谱法与CE结合,将有利于简化CE对糖蛋白的富集过程^[16]^。

基于上述问题,本研究首先制备了3-氨基苯硼酸(APBA)修饰的纳米金(AuNPs@APBA),利用离子吸附作用将AuNPs@APBA吸附到毛细管内壁,制得pH响应型涂层毛细管柱,并以此涂层毛细管柱为分离通道,实现了糖蛋白的在线富集与分离。

## 1 实验部分

### 1.1 仪器与试剂

P/ACE MDQ毛细管电泳仪(美国贝克曼库尔特有限公司); TENSOR 27傅里叶红外光谱仪(德国布鲁克公司);熔融石英毛细管柱(60.2 cm×75 μm)(永年县锐沣色谱器件有限公司); MalvernZen3600激光粒度仪和Zeta电位仪(英国马尔文公司); PB-10 pH计(德国赛多利斯股份公司)。

氯金酸(HAuCl_4_·4H_2_O,纯度99.5%,上海阿拉丁试剂有限公司);柠檬酸钠(纯度99%,天津市盛奥化学试剂公司); APBA(纯度98%,北京百灵威科技有限公司);柠檬酸(分析纯,西安化学试剂厂);海美溴铵(HDB,纯度≥94.0%,西格玛化学试剂有限公司);牛血清白蛋白(BSA,纯度≥98.0%,上海源叶生物技术有限公司);卵清蛋白(OVA,纯度95.0%,北京索莱宝生物科技有限公司);二甲基亚砜(DMSO,分析纯,天津市天力化学试剂有限公司);所有实验用水均为去离子水(杭州娃哈哈集团有限公司)。

### 1.2 AuNPs@APBA的制备

采用柠檬酸钠还原法制备AuNPs。精密量取3.5 mL氯金酸水溶液(25.4 mmol/L)和96.5 mL去离子水于配有磁力搅拌子的250 mL三颈烧瓶中,在油浴中加热并搅拌至沸腾;加大搅拌速度,快速加入10 mL柠檬酸钠水溶液(38.8 mmol/L),待溶液颜色变为透亮的酒红色后,继续搅拌10 min;之后在搅拌状态下将溶液自然冷却至室温,并在室温下继续搅拌15 min,即得到AuNPs。移取5 mL AuNPs分散液于15 mL烧杯中,加入0.25 mg APBA,在室温下搅拌反应6 h,即得AuNPs@APBA,于4 ℃冰箱中保存。

### 1.3 AuNPs@APBA涂层毛细管柱的制备

取熔融石英毛细管柱,分别用1 mol/L NaOH溶液、去离子水、1 mol/L HCl溶液、去离子水冲洗60、5、15、5 min,以活化毛细管柱。

在137.9 kPa压力下,向活化后的毛细管柱内通入质量分数为0.1%的HDB水溶液,持续2 min,停留5 min,之后吹去未吸附的溶液,获得HDB涂覆的正电荷涂层毛细管柱;在137.9 kPa压力下,将AuNPs@APBA分散液(1.14 nmol/L)注入上述毛细管柱,持续2 min,在柱内停留5 min后吹去未吸附的AuNPs@APBA,重复上述操作进行二次涂层,即得到AuNPs@APBA涂层毛细管柱。

### 1.4 样品制备

分别精密称取0.5048 g OVA和0.5013 g BSA,加入去离子水溶解,并分别定容于50 mL容量瓶中,混匀,即得质量浓度为1.0 mg/mL的OVA和BSA样品溶液。

将新鲜鸡蛋清样品用去离子水稀释20倍,于室温下搅拌10 min,在4 ℃、10000 r/min下离心10 min,所得到的上清液即为鸡蛋清母液;用去离子水对鸡蛋清母液进行稀释,即得到不同稀释倍数的鸡蛋清样品。

### 1.5 糖蛋白的在线富集与分离

基于pH响应涂层柱的CE法:在137.9 kPa压力下,用磷酸盐缓冲液(PBS, 10 mmol/L, pH 8)冲洗AuNPs@APBA涂层毛细管柱5 min,在20 kV电压下预电泳2 min,之后将糖蛋白样品在3.4 kPa压力下进样,进样时间5 s;在137.9 kPa压力下,用PBS(10 mmol/L, pH 8)冲洗AuNPs@APBA涂层毛细管柱1 min,在34.5 kPa压力下向毛细管柱中注入PBS(10 mmol/L, pH 3),持续10 s,随后在20 kV电压下、pH为3的PBS(10 mmol/L)中进行分离(图1)。实验过程中柱温设置为25 ℃,紫外检测波长设置为214 nm。

**图1:**
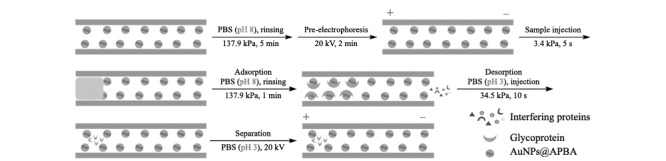
糖蛋白的在线富集与分离示意图

普通电泳法:在137.9 kPa压力下,用PBS(10 mmol/L, pH 7)冲洗裸柱5 min,之后将糖蛋白样品在3.4 kPa压力下进样,进样时间5 s。实验过程中电压设置为20 kV,柱温设置为25 ℃,紫外检测波长设置为214 nm。

## 2 结果与讨论

### 2.1 AuNPs与AuNPs@APBA的表征

利用柠檬酸钠还原法制备的AuNPs表面含有羧基,而APBA的表面含有氨基,二者通过静电相互作用结合。对AuNPs和AuNPs@APBA进行红外光谱(FT-IR)表征,结果如图2所示,AuNPs@APBA在1358 cm^-1^处出现的吸收峰归属于苯硼酸中的B-O,证实了AuNPs@APBA的成功制备。对AuNPs和AuNPs@APBA进行粒径和Zeta电位表征,结果表明,AuNPs的粒径为(29.33±1.26) nm, Zeta电位为(-69.50±1.12) mV; AuNPs@APBA的粒径为(37.03±0.30) nm, Zeta电位为(-48.10±0.41) mV;在修饰了APBA后,AuNPs的粒径和Zeta电位均增大,表明APBA已成功修饰到AuNPs的表面。此外,根据测得的AuNPs粒径及文献[17],计算得到本实验所制备的AuNPs浓度为1.14 nmol/L。

**图2:**
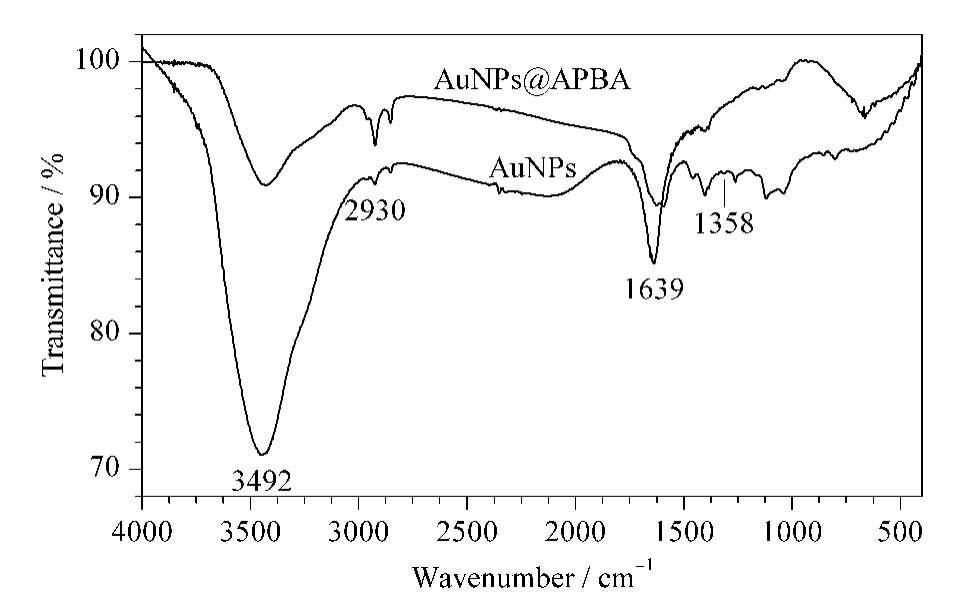
AuNPs和AuNPs@APBA的FT-IR图

### 2.2 AuNPs@APBA涂层毛细管柱的表征

本研究利用DMSO来验证AuNPs@APBA涂层毛细管柱的性能。实验结果表明,经HDB涂覆后,DMSO的出峰时间由5.05 min延长至10.88 min,电渗流(EOF)值从2.978×10^-2^cm^2^/(V·s)降低至1.383×10^-2^cm^2^/(V·s),由此可知毛细管柱内壁已成功修饰正电荷涂层;经AuNPs@APBA涂覆后,DMSO的出峰时间由10.88 min缩短至9.70 min, EOF值增大至1.552×10^-2^cm^2^/(V·s),该结果表明AuNPs@APBA已成功涂覆至毛细管柱内壁。

### 2.3 解吸附条件的考察

在pH为8的条件下,糖蛋白能够与AuNPs@APBA表面的硼酸基团结合形成硼酸酯,从而被吸附;当pH为3时,硼酸酯发生解离,糖蛋白被释放。基于上述原理,AuNPs@APBA涂层毛细管柱可实现糖蛋白的选择性在线富集与分离。在糖蛋白的解吸附过程中,PBS的注入压力会对糖蛋白的富集分离效果产生影响。本实验以稀释1×10^6^倍的鸡蛋清样品为考察对象,优化了解吸附过程中PBS(pH 3)的注入压力。由图3可知,当PBS(pH 3)的注入压力分别为137.9 kPa和103.4 kPa时(注入时间均为10 s),电泳峰的基线不稳且存在拖尾现象;当注入压力降低至34.5 kPa时,电泳峰基线平稳,样品峰的峰面积可达17226 mAU·ms,理论塔板数可达134485 个/m,对称因子为0.991,可见适当减小注入压力有助于提高糖蛋白样品的富集分离效果。分析其原因,注入压力过大可能会导致样品的解吸附速度产生差异,从而会导致电泳峰基线不稳及拖尾现象等。因此,本实验最终选择在34.5 kPa压力下注入PBS(pH 3),注入时间为10 s。

**图3:**
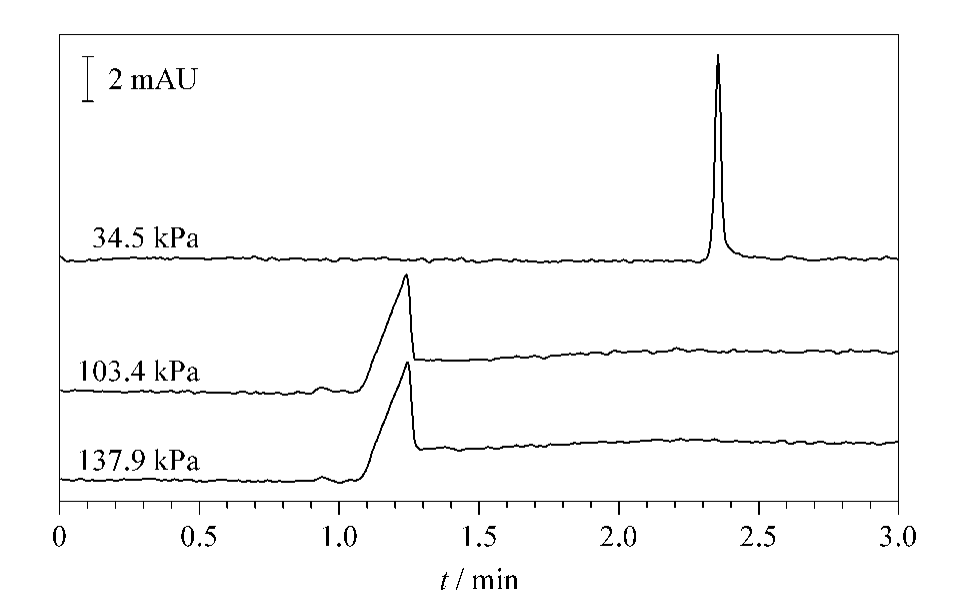
鸡蛋清样品(稀释1×10^6^倍)在不同注入压力下的电泳图

### 2.4 OVA和BSA的在线富集与分离

利用本文所建方法对OVA(1.0 mg/mL)和BSA(1.0 mg/mL)样品溶液进行在线富集与分离,并通过峰面积的增大倍数来评价富集效果,其中峰面积的增大倍数为样品在基于pH响应的AuNPs@APBA涂层柱和基于普通电泳法的裸柱中所得峰面积之比。如图4a和4b所示,经AuNPs@APBA涂层毛细管柱分离后,OVA和BSA的峰面积分别增大了26.46倍和8.47倍,表明本文所制备的AuNPs@APBA涂层毛细管柱对糖蛋白的富集效果优于普通蛋白(BSA),具有选择性富集作用;此外,与使用普通电泳法的裸柱相比,pH响应涂层柱的富集效果明显更好。与本课题组^[16]^前期制备的APBA-聚甲基丙烯酸缩水甘油酯微球(PGMA@APBA)涂层毛细管柱相比,OVA的峰面积增大了约20倍,这可能是因为与PGMA微球(粒径(138.00±0.20) nm)相比,AuNPs的比表面积更大,能够吸附更多的APBA分子,有助于提高糖蛋白的富集效果。

**图4:**
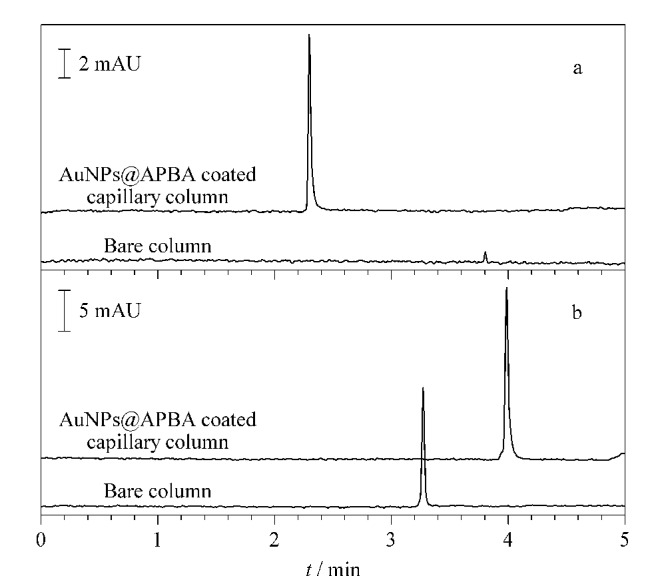
(a)OVA和(b)BSA经AuNPs@APBA涂层毛细管柱和裸柱分离后的电泳图

### 2.5 重复性和稳定性考察

以OVA样品溶液(1.0 mg/mL)为例,利用本文所建方法进行在线富集与分离,测定OVA的电泳峰面积;于1 d内平行测定5次,计算日内精密度;连续测定6 d,计算日间精密度。实验结果表明,OVA电泳峰面积的日内和日间精密度分别为2.2%(*n*=5)和3.0%(*n*=6),方法重复性良好。在使用过程中,缓冲液的冲洗或其他因素都容易造成毛细管柱的涂层脱落,使其稳定性变差,故本实验对AuNPs@APBA涂层毛细管柱的稳定性进行了考察。实验结果表明,当AuNPs@APBA涂层毛细管柱连续运行100次(总计33~67 h)时,其对鸡蛋清样品(稀释1×10^6^倍)中的糖蛋白仍具有一定的富集效果(第100次时峰面积仍达6505 mAU·ms),说明本工作所制备的AuNPs@APBA涂层毛细管柱稳定性良好,能够满足实际应用需求。

### 2.6 实际样品中糖蛋白的富集与分离

以不同稀释倍数下的鸡蛋清为实际样品,考察本方法对鸡蛋清样品中痕量糖蛋白的富集分离效果。不同稀释倍数下鸡蛋清样品的电泳图如图5所示,将鸡蛋清样品稀释20倍后,与使用普通电泳法的裸柱相比,其在AuNPs@APBA涂层毛细管柱上的峰面积增大了1.15倍(图5a);将鸡蛋清样品稀释2×10^3^倍后,与使用普通电泳法的裸柱相比,其在AuNPs@APBA涂层毛细管柱上的峰面积可增大至33.93倍(图5b);将鸡蛋清样品稀释1×10^6^倍后,裸柱(普通电泳法)已无法检测到糖蛋白,而AuNPs@APBA涂层毛细管柱对糖蛋白的富集分离效果仍非常明显,峰面积为10469 mAU·ms(图5c),且整个富集分离过程可在3 min内完成。实验结果证明,该方法可用于实际样品中痕量糖蛋白的在线富集与分离。此外,对于稀释了20倍和2×10^3^倍后的鸡蛋清样品,通过本方法富集后的糖蛋白峰面积相差不大,推测可能与毛细管内壁所吸附的AuNPs@APBA数量有关:当涂覆至毛细管内壁的AuNPs@APBA数量一定时,毛细管柱所能捕获的糖蛋白数量存在上限。因此,对糖蛋白含量较高的样品进行富集分离时,需进一步提高毛细管柱内壁单位面积上硼酸基的数量,以吸附更多的糖蛋白分子。

**图5:**
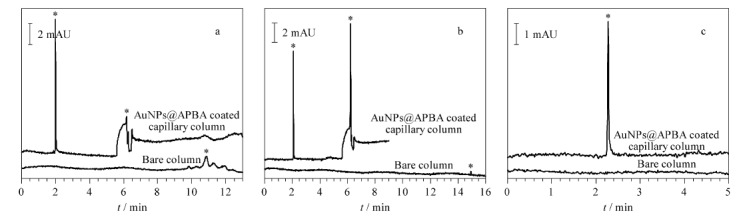
不同稀释倍数下的鸡蛋清样品经AuNPs@APBA涂层毛细管柱和裸柱分离后的电泳图

## 3 结论

本研究通过静电自组装法制备了AuNPs@APBA涂层毛细管柱,建立了一种基于pH响应涂层柱的糖蛋白在线富集与分离方法。该方法利用APBA与糖蛋白之间的特异性相互作用,并通过改变pH条件实现了糖蛋白的在线富集与分离。该方法操作简单,省时,高效,弥补了化学键合涂层柱的不足,但利用本方法所涂覆的APBA数量有限,限制了该涂层柱对高含量糖蛋白样品的富集效果,未来可考虑采用含有多个硼酸基的聚合物刷对毛细管进行涂覆,通过改变聚合物刷的长度来调控硼酸基数量,从而满足不同含量糖蛋白样品的富集要求。
